# Relationship of daily total somatic cell output with somatic cell concentration and clinical mastitis

**DOI:** 10.3168/jdsc.2020-0065

**Published:** 2021-06-23

**Authors:** H.P. Lehew, C.D. Dechow

**Affiliations:** Department of Animal Science, Pennsylvania State University, University Park 16802

## Abstract

Somatic cell count (SCC) measures the concentration of somatic cells in milk and is used as a mastitis diagnostic tool. It is plausible that variation in milk yield could alter the relationship between SCC and mastitis status. Our objective was to evaluate total daily SCC output as a predictor of clinical mastitis. Data included 37,035 test-day records from 4,179 lactations of 1,679 cows and 1,286 mastitis events from an experimental herd. Daily total SCC was derived by multiplying SCC by daily milk yield in milliliters and transformed to daily total somatic cell score (DTSCS) via a log_2_ transformation. Milk yield, SCS, and DTSCS were evaluated with mixed models that included the proximity of a mastitis event to the test date and days in milk as the main fixed effects. A second series of logistic regression was conducted that considered mastitis (1 = mastitis occurred during a test interval; 0 = no mastitis) as the dependent variable with milk yield, SCS, and DTSCS the main independent effects. Least squares means for test dates associated with mastitis-free lactations were 2.43 and 2.25 for SCS and DTSCS, respectively. The corresponding values were 5.96 for SCS and 5.66 for DTSCS for the week of a mastitis event. Whereas SCS declined rapidly in early lactation and then increased steadily thereafter, DTSCS was lowest in early lactation and increased by a proportionally smaller amount throughout lactation. Including both SCS and DTSCS in the same model improved the logistic regression model fit over a model with SCS only. Dilution effects from milk yield influence SCS, and consideration of DTSCS in management and genetic selection schemes could improve mastitis detection and resistance.

## Highlights

• Measures of somatic cells in milk typically reflect concentration of cells but not total cell output.
• Milk yield has a dilution effect, and elevated concentration of somatic cells in early and late lactation partly reflects lower yield.
• Consideration of both somatic cell concentration and total daily somatic cell output may improve mastitis detection.

## Graphical Abstract

**Figure:**
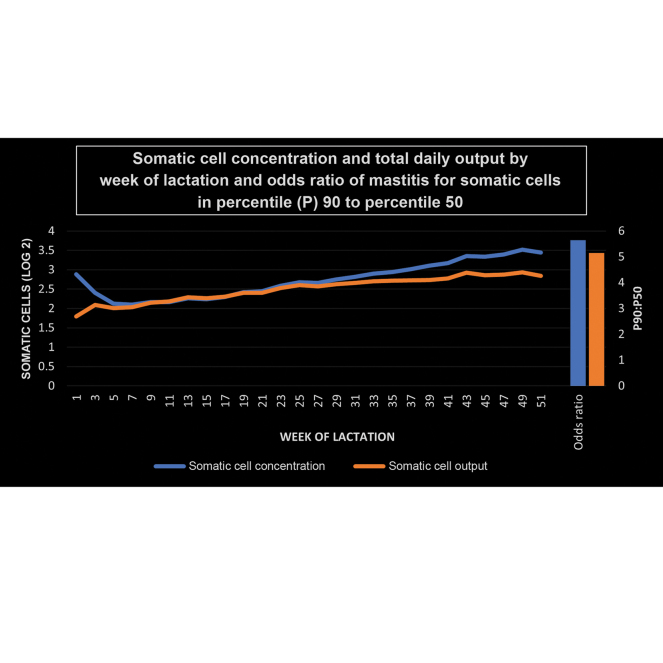
**Summary:** Somatic cell count concentration has a well-established decline in early lactation followed by a steady increase as lactation progresses. However, it appears that elevated early- and late-lactation somatic cell concentration is partly explained by lower milk yield at the ends of lactation. The total number of somatic cells output in milk is lowest in early lactation followed by a smaller rise than observed for somatic cell concentration. Elevated somatic cell concentration and somatic cell output were both associated with increased odds of mastitis during a milk testing interval. The odds of mastitis was approximately 5-fold higher for cows in the 90th percentile for somatic cell concentration or output than for cows in the 50th percentile.

Somatic cell count and its log_2_ transformation (SCS) has been used as an indicator of mastitis and in genetic selection programs to increase resistance to mastitis (Schutz, 1994). Genetic trends indicate that genetic merit for SCS has improved for US Holsteins since 2000 and has been stable over the last decade for US Jerseys (CDCB, 2020). Various studies have compared alternative measures of SCS in relationship to mastitis incidence. de Haas et al. (2008) evaluated associations of average SCC over lactation periods of varying lengths, presence of SCC spikes, or SCC patterns with mastitis incidence and concluded that multiple SCC traits could be more effective than using only lactation-average SCS in selection to improve udder health. The standard deviation of SCC and presence of test-day SCC >500,000 cells/mL were reported to be strongly correlated with clinical mastitis (Urioste et al., 2012), and using those measures in addition to SCS average from early lactation improved prediction accuracy of mastitis breeding values (Koeck et al., 2012). Considering traits such as consecutive test dates with SCC above a given threshold was also recommended as a strategy to improve selection for udder health (Kirsanova et al., 2019). An analysis of SCC distribution in relationship to the presence of pathogens on a per-quarter basis suggests that there is more information to be extracted from SCC than current practices, which rely mostly on averages or cutoffs for SCC and SCS (ten Napel et al., 2009).

There are different ways to express SCC and different manners in which SCC is used to evaluate mastitis resistance; however, most genetic selection and mastitis management programs rely on the concentration of somatic cells as opposed to the total number of somatic cells that a cow outputs daily. It is plausible that variation in milk yield could alter the relationship between SCS and mastitis status. Contrasting SCC and milk yield in Holsteins and Jerseys helps demonstrate the dilution effect for cows with higher yield. Jersey herds in the upper Midwest of the United States had an average bulk tank SCC of 246,000 cells/mL compared with 204,000 cells/mL for Holstein herds (AgSource, 2020). However, milk yield was higher in Holstein herds (33.0 L/cow per day) than in Jersey herds (23.9 L/cow per day); the result is that Holsteins produced more total somatic cells (6.7 billion/d) than Jerseys (5.9 billion/d) despite higher SCC in Jersey herds. Our study aimed to determine relationships among SCS, milk yield, and total daily somatic cell output as predictors of clinical mastitis.

Records from Holsteins in lactation 1 through 7 from The Pennsylvania State University dairy herd from January 2000 through June 2018 were used for this evaluation; very few records (0.19% of total from 8 cows) were from later than lactation 7 and therefore were not considered. Data included 37,035 monthly test-day records from 4,179 lactations of 1,679 cows. Only test days occurring within 1 yr of calving were retained, and there was no restriction on the number of test days required per cow lactation, as cows with mastitis might be culled and have few observations. Test-day records of SCS and milk yield were retrieved from Dairy Comp 305 (Valley Ag Software) along with mastitis events. A mastitis event in this herd refers to clinical mastitis identified via visual examination of foremilk stripped from each quarter followed by a California Mastitis Test or other acute symptoms (Penn State College of Agricultural Sciences, 2011). Following diagnosis by the milking staff, the event is recorded in a parlor log and the sample is cultured and entered into Dairy Comp 305 after the cow is examined by the herd manager. Daily total SCC (**DTSCC**) was derived by multiplying SCC by daily milk yield in milliliters; we assumed that 1 kg of milk was equivalent to 1,000 mL. We then normalized DTSCC to create daily total SCS (**DTSCS**) using a log_2_ transformation of DTSCC in billions plus 1; a log_2_ transformation and constant of 1 were selected to facilitate an easier interpretation of DTSCS as a 1-unit increase in DTSCS represents a doubling of DTSCC, and the constant of 1 sets DTSCS to 3 for a cow producing 40 L of milk with an SCS of 3.

Mastitis events (n = 1,286) were merged to the nearest test date. Test dates were classified as follows to derive the mastitis proximity categories: 8 to 14 d before mastitis (−2 wk; n = 268), 7 to 3 d before mastitis (−1 wk; n = 184), 2 d before to 2 d after mastitis (0 wk; n = 238), 3 to 7 d after mastitis (+1 wk; n = 254), and 8 to 14 d after mastitis (+2 wk; n = 342). Cows treated for mastitis still have their milk weighed and sampled on a test date in this herd even though the milk is subsequently discarded. Also included were test days that occurred during a lactation that had mastitis, but not within 14 d (>|2| wk; n = 9,123), and that were associated with a mastitis-free lactation (none; n = 26,626).

Milk yield, SCS, and DTSCS were evaluated with the MIXED procedure of SAS (v. 9.4, SAS Institute Inc.) with mastitis proximity (−2 wk to +2 wk, >|2|, none), biweekly classes of DIM, and lactation number as fixed effects; cow identification, test date, the interaction of biweekly DIM class within lactation group (1, ≥2), and residual error were fit as random effects. Least squares means (**LSM**) were derived for mastitis proximity and biweekly DIM with a Tukey adjustment to determine differences. As there are many more observations in lactation 1 (14,207) than in lactation 7 (120), the OM (observed margins) option of the LSMEANS statement in SAS was implemented so that reported LSM reflect the relative frequency of different lactations as opposed to equal weighting. Differences were considered significant at *P* < 0.05.

A second series of analyses was conducted that considered clinical mastitis as the dependent variable. Mastitis (1 = clinical mastitis occurred during the monthly test interval; 0 = no mastitis) was evaluated as a binary variable with logistic regression using the GLIMMIX procedure of SAS. Regression on milk yield, SCS, and DTSCS was fit individually and jointly to determine associations with the odds of mastitis. Additional effects included lactation group (lactation 1, 2, and ≥3), year, month, and biweekly DIM class. Attempts to fit cow as a random effect did not result in convergence, indicating that there was an insufficient amount of variation associated with cow to facilitate detection given our sample size. The resulting regression coefficients for milk yield, SCS, and DTSCS were used to derive odds of mastitis for a cow in lactation ≥3, the first 2 wk of lactation, and milk yield, SCS, or DTSCS at the 10th, 25th, 50th, 75th, and 90th percentiles. The best-fitting model was declared the one that minimized the Bayesian information criterion (**BIC**).

Average daily milk yield was 33.6 L in lactation 1, 39.2 L in lactation 2, and 42.0 L in lactation ≥3, with average SCC of 132,565 in lactation 1, 211,663 in lactation 2, and 358,905 in lactation ≥3. Average SCS was 2.05, 2.47, and 3.05 in lactations 1, 2, and ≥3, respectively. The average DTSCC in lactations 1, 2, and ≥3 was 4.1 billion, 7.3 billion, and 13.4 billion, respectively, whereas average DTSCS was 1.74, 2.37, and 3.03, respectively. The random test-date effects were used to examine yearly trends. The annual trend in milk yield declined from 2000 to 2011 (−0.13 kg/yr) and then increased steadily until 2018 (+0.22 kg/yr). Both SCS and DTSCS declined at a rate of −0.01 point/yr from 2000 to 2018.

Mastitis proximity had a significant association with milk yield, SCS, and DTSCS, with LSM displayed in Figure 1. The trends for LSM of SCS and DTSCS were similar, with estimates being lowest for records from mastitis-free lactations (2.43 ± 0.06 and 2.25 ± 0.04, respectively) and highest for records from 0 wk (i.e., 2 d before to 2 d after test day; 5.96 ± 0.11 and 5.66 ± 0.10, respectively). Shook et al. (2017) reported a mean SCS of 2.59 for cows with no bacterial growth from a milk culture, which is similar to our result of 2.43 for mastitis-free lactations. Contagious pathogens (4.07) and environmental pathogens (3.66) both elevate SCS according to Shook et al. (2017). Our SCS during 0 wk was higher than those values, which could reflect that we were evaluating clinical mastitis, whereas bacterial growth can occur for both clinical and subclinical mastitis.

**Figure 1:**
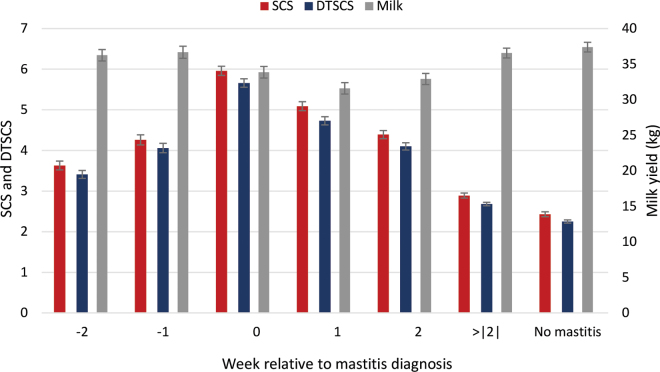
Least squares means (±SE) of test-day SCS, daily total SCS (DTSCS), and milk yield by proximity of test date to mastitis diagnosis.

The results in Figure 1 indicate an increase in SCS of 3.53 and an increase in DTSCS of 3.41 during the week of a mastitis event (0 wk) compared with lactations with no mastitis; this is equivalent to an 11.6-fold increase in SCC and a 10.6-fold increase in DTSCC. As expected, the relationship of mastitis and milk yield was largely opposite that of mastitis and SCC, with milk yield being highest for mastitis-free records (37.7 L). The nadir of milk yield was observed for records from 1 wk after mastitis (31.6 L), which was significantly less (*P* < 0.01) than 0 wk (33.9 L); the LSM for +2 wk (32.9) was not significantly different from that for 0 wk or +1 wk.

There is a potential source of bias when evaluating relationships between SCC and mastitis if SCC or SCS are used to diagnose mastitis and treat mastitis by herd management. This would be particularly evident for test dates that occur before a mastitis event. We would expect a larger number of records when a test date occurs before mastitis (−2 wk and −1 wk) if SCS was used for mastitis treatment decisions, but that was not apparent in the data. Based on this and communication from herd management that SCS is used to make culling decisions in this herd more often than mastitis treatment decisions, we believe the results in Figure 1 are relatively unbiased by use of SCS for mastitis diagnosis. Nevertheless, it is likely that the relationship between SCS and clinical mastitis is biased upward to some degree when SCS is used to diagnose mastitis.

The LSM of SCS and DTSCS for the biweekly DIM effect are presented in Figure 2, with labels on the x-axis representing the midpoint of each biweekly DIM period in weeks of lactation. The general lactation curve for SCS of rapid decline in early lactation followed by a steady rise until the end of lactation has been previously reported (Wiggans and Shook, 1987; De Haas et al., 2002; Dechow and Goodling, 2008; Hagnestam-Nielsen et al., 2009; ten Napel et al., 2009); cows thought to be free from infection also show this general pattern but to a lesser degree (Harmon, 1994). Despite the high initial values of SCS, it is apparent from the DTSCS results that total somatic cell output is lowest in early lactation. The early-lactation decline in SCS appears to be largely the result of a dilution effect of higher milk yield. The early-lactation decrease has been previously reported, and elevated SCS near calving does not necessarily indicate mammary infection (Dohoo and Meek, 1982). On the other hand, the increase in SCC as lactation progressed was primarily the result of higher SCC concentration, and previous reports suggest that this is primarily due to an increase in IMI during lactation (Dohoo and Meek, 1982; Harmon, 1994).

**Figure 2:**
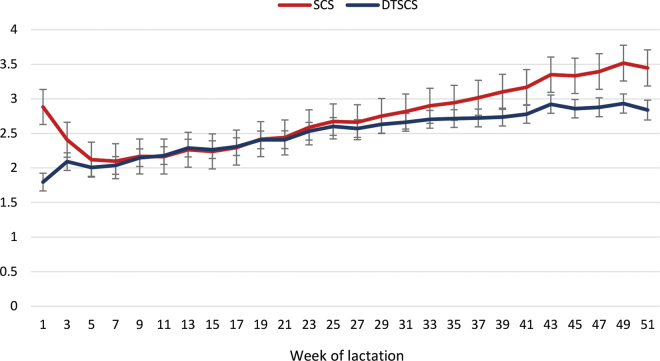
Least squares means (±SE) of SCS and daily total SCS (DTSCS) by week of lactation.

Odds ratios and 95% confidence intervals for mastitis at various percentiles of SCS, DTSCSS, and milk yield are reported in Table 1. Cows that had high SCS or DTSCS had higher odds of being diagnosed with clinical mastitis. The estimated probability of a mastitis event was 2.09, 2.99, 5.20, 10.74, and 22.67% at the 10th, 25th, 50th, 75th, and 90th percentiles of SCS, respectively, for a cow in lactation ≥3 in the first 2 wk of lactation. The mastitis probabilities for SCS correspond to an odds ratio of 5.35:1 for cows in the 90th percentile relative to the 50th percentile, whereas the odds ratio for the 10th to 50th percentiles was 0.39:1. For DTSCS, the odds ratios for the 90th and 10th percentiles relative to the 50th were similar (4.90:1 and 0.42:1, respectively) to those observed for SCS but slightly lower in magnitude. Shook et al. (2017) reported that a 1-point increase in SCS was associated with a 9.1% increase in IMI. Our observed increase in clinical mastitis events was less (4.1% per 1-point increase in SCS from the 10th to 90th percentiles; Table 1), but this is not surprising because our observations are of clinical mastitis only. Our observed relationship of clinical mastitis with SCS and DTSCS is also not linear, with the percentage rising more rapidly as SCS and DTSCS increase. An increased pathogen shedding intensity among cows with IMI as SCS increases has been previously reported (Hamel et al., 2021); this could be partly responsible for the nonlinear relationship of SCS and DTSCS with clinical mastitis.

**Table 1:** Percentile value (PV) and odds ratios (OR) for mastitis in the 10th, 25th, 50th, 75th, and 90th percentiles of SCS, daily total SCS (DTSCS), and milk yield relative to the 50th percentile with lower (L95) and upper (U95) 95% confidence limits

Percentile	SCS				DTSCS				Milk			
	PV	OR	L95	U95	PV	OR	L95	U95	PV	OR	L95	U95
10	0.30	0.39	0.41	0.37	0.33	0.42	0.44	0.40	25.00	1.29	1.39	1.21
25	1.00	0.56	0.58	0.54	0.96	0.58	0.60	0.56	31.36	1.14	1.18	1.10
50	2.10	1.00	NA^1^	NA	1.99	1.00	NA	NA	37.73	1.00	NA	NA
75	3.60	2.19	2.10	2.29	3.37	2.07	1.99	2.16	45.00	0.86	0.83	0.90
90	5.30	5.35	4.88	5.86	5.00	4.90	4.49	5.34	51.82	0.75	0.70	0.81

1 Not applicable.

The odds ratios for milk yield indicated that cows with the highest milk yield were least likely to have mastitis, but the effect was not as strong as those observed for SCS or DTSCS. Higher milk yield is reported to result in higher odds of mastitis in the subsequent lactation (Rupp et al., 2000), which suggests that the association of milk yield and mastitis we observe likely reflects the effect of mastitis on milk yield as opposed to the effect of milk yield on mastitis.

When SCS, DTSCS, and milk yield were included as independent effects separately, the model that included SCS was the best fit (BIC = 8,997.4), followed by the model including DTSCS (BIC = 9,034.8) and milk yield (BIC = 10,394.6). Only the effect of SCS was significant when SCS, DTSCS, and milk yield were included in the same model. However, both covariates were significant for models including SCS and DTSCS, SCS and milk yield, or DTSCS and milk yield; the model containing SCS and DTSCS had the best fit of the multivariate models (BIC = 8,999.6), followed by SCS and milk yield (BIC = 9,000.1). When interactions among effects were included, the best-fitting model was that containing SCS and DTSCS (BIC = 8,996.4), followed by that containing SCS and milk yield (BIC = 9,004.6). This suggests that consideration of both SCS and DTSCS could help in mastitis detection but that SCS is the best measure if restricted to one measure.

In conclusion, this study demonstrates that variation in milk yield alters the concentration of somatic cells in milk. Consideration of DTSCS in addition to traditional measures of somatic cell concentration (SCC and SCS) may improve detection of mastitis and selection for mastitis resistance.
